# Severe Influenza With Invasive Pulmonary Aspergillosis in Immunocompetent Hosts: A Retrospective Cohort Study

**DOI:** 10.3389/fmed.2020.602732

**Published:** 2021-01-18

**Authors:** Yishan Duan, Xinyan Ou, Yusha Chen, Binmiao Liang, Xuemei Ou

**Affiliations:** ^1^Department of Respiratory Medicine, Sichuan University West China Hospital, Chengdu, China; ^2^College of Computer Science, Chongqing University, Chongqing, China

**Keywords:** severe influenza, aspergillosis, prognostic factors, immunocompetent hosts, excessive inflammatory response

## Abstract

**Background:** Influenza was an independent risk factor for invasive pulmonary aspergillosis (IPA). In light of increasing incidence and mortality of influenza associated aspergillosis, our study summarized risk factors, clinical characteristics, and prognostic factors of developing aspergillosis in immunocompetent hosts with influenza to further screen high-risk population and improve outcome.

**Methods:** We reviewed the patient characteristics, laboratory examinations, radiological imaging, and microbiology data of 72 influenza patients with IPA and 84 influenza patients without IPA admitted to West China Hospital.

**Result:** Our study shown that aspergillosis co-infection increased overall mortality of severe influenza from 22.6 to 52.8%, along with higher white blood count (WBC) (10.9 ± 5.0 vs. 8.4 ± 3.3, *P* = 0.016), Neutrophiles (9.5 ± 5.0 vs. 7.0 ± 3.8, *P* = 0.023), procalcitonin (PCT) (8.6 ± 15.9 vs. 1.2 ± 2.1, *P* = 0.009), and a lower CD4^+^ T cell count (189.2 ± 135.3 vs. 367.1 ± 280.0, *P* = 0.022) in death group. No impact of age, gender, underlying diseases, immunosuppressive agents and steroids use, CD4^+^ T cell count on incidence of influenza associated aspergillosis was observed. But influenza associated aspergillosis cases mostly accompanied with more H1N1 subtype (91.7 vs. 79.8%, *P* = 0.037) and higher level of C-reactive protein (CRP) (117.6 ± 88.1 vs. 78.5 ± 75.2, *P* = 0.017) and interleukin 6 (IL-6) (133.5 ± 149.2 vs. 69.9 ± 100.0, *P* = 0.021) than those without aspergillosis.

**Conclusion:** Aspergillosis co-infection in severe influenza patients can lead to a significant increased mortality, which was associated with severe respiratory failure due to mixed infection and immunosuppression. Pulmonary excessive inflammatory response was related with IPA co-infection.

## Introduction

Increasing aspergillosis co-infection have been reported as a common complication of severe influenza and became an important cause of increased mortality among severe influenza patients in recent years (1–7). A sharp increase in reported cases occurred after the 2009 H1N1 influenza pandemic and 128 influenza associated aspergillosis cases have been published up to June 2018 in literature review of cases and case series (6), and the incidence rate of invasive pulmonary aspergillosis (IPA) reached up to 19% in the setting of severe influenza (3). With a deeper understanding of invasive fungal diseases (IFDs), the updated and revised definitions of IFDs expanded host factors for probable IFDs and new criteria for proven IFDs is applicable to patients even without host factors (8). Significant increased aspergillosis co-infection in the setting of severe influenza suggested that severe influenza was probably one of host factors. The bigger point is that the occurrence of IPA among severe influenza patients usually entails a poorer prognosis. The overall mortality of influenza associated aspergillosis reported in systematic reviews fluctuated between 40 and 60%, which was generally thought to be much higher than mortality of severe influenza patients with other co-infections or without co-infections (1, 3, 5, 6).

IPA typically occurs in immunocompromised hosts. But majority of reported influenza associated aspergillosis cases did not have classic host factors for IPA and influenza was observed to be independently associated with IPA in various studies (5, 6, 9, 10). Glucocorticoids was generally considered to be an important cause of aspergillus co-infection in patients with severe influenza, as glucocorticoids was frequently used in severe influenza patients with septic shock or acute respiratory distress syndrome (ARDS) (11, 12). Moreover, male sex, age, and comorbidities (including cancer, chronic obstructive pulmonary disease (COPD), severe hepatic insufficiency, and renal failure) have also been reported as risk factors for aspergillosis co-infection and were likely associated with increased mortality of aspergillosis group in some studies with small sample sizes (1, 2, 4, 6, 13). However, many young patients without underlying disease and glucocorticoid use were observed to develop IPA in the setting of severe influenza. There are still controversies in potential risk factors of developing IPA in the setting of severe influenza because the sample sizes of researches on these issues were too small. Moreover, there was still lack of detailed studies on target clinical manifestations, imaging features, and poor prognosis of influenza associated aspergillosis.

Our study enrolled a total of 156 severe influenza patients, systematically and comprehensively analyzed differences in patient characteristics, clinical features, and outcome parameters between severe influenza patients with and without aspergillosis. Besides, we also attempt to conduct risk evaluation of poor prognosis among influenza associated aspergillosis cases and explore the impact of clinical diagnosis and treatment on outcomes of influenza associated aspergillosis to identify the better diagnostic and management strategy.

## Methods

### Study Population

Our study consisted of 72 patients diagnosed with severe influenza with IPA in West China Hospital of Sichuan University from December 2018 to March 2019, and 84 severe influenza patients in the same period matched by age and gender. Among them, 75 patients were admitted to Intensive Care Unit (ICU), 81 patients were admitted to respiratory department or infectious diseases department. Patients < 18 years were excluded.

### Definition of Severe Influenza

Diagnostic criteria for influenza include clinical symptoms of influenza and at least one positive assay for the following pathogens detection: real time polymerase chain reaction; viral isolation; viral antigen detection (1).

Severe influenza refers to patients with symptoms of lower respiratory tract infection or severe complications (pneumonia, septic shock, multiple organ dysfunction, and so on) (14, 15).

### Diagnosis of IPA

The IPA diagnostic criteria consists of host factors, clinical features, and mycological evidence on the basis of EORTC/MSG criteria (16). Consensus that influenza was independently associated with IPA has been reached in previous studies and autopsy series of severe influenza indicated that strict interpretation of the host factors for IPA contributed to missed diagnosis of IPA (17, 18), so we used a modified EORTC/MSG criteria in which no specific host factor was required (1). Clinical features included new infiltrates on the chest X-ray or computed tomography (CT) scan, as well as clinical symptoms including fever or dyspnea, hemoptysis, and/or wheezing. Mycological evidence included positive aspergillus cultivation, galactomannan (GM) antigen detected in serum (i.e., ≥0.5) or bronchoalveolar lavage fluid (BAL, optical density ≥ 1.0) (3, 16). According to EORTC/MSG criteria, patients were classified into three types of IPA (proven IPA, probable IPA, and possible IPA) (16). In order to fully exclude aspergillus colonization, both clinical symptoms and new infiltrates in pulmonary imaging are required for patients with only positive culture for aspergillus (19). All the IPA diagnosis was ultimately made by two senior respiratory physicians on basis of comprehensive consideration of clinical symptoms, imaging findings, and microbial evidence.

### Data Collection

Data were recorded from medical records among all cases, including demographics, underlying diseases, immunosuppressive agents and steroids use, cumulative doses of steroids. Besides, the initial clinical symptoms and outcome parameters (oxygenation index, respiratory support method, ICU admission, and mortality) were also included into our study. Moreover, we reviewed laboratory examinations, radiological imaging (pulmonary X-ray/CT), microbiology data (bacterial, fungus, and viral examinations) during hospitalization. The lung imaging findings were divided into four patterns: consolidation, ground-glass opacity (GGO), nodular, and cavity sign (20, 21). (If there were more than two types abnormal imaging patterns in pulmonary X-ray/CT, the major one should be selected as the main imaging characteristic).

### Statistical Analysis

The analysis was performed with SPSS 24.0 (SPSS Inc., Chicago, IL, USA). Qualitative variables were expressed as counts and frequencies and quantitative variables were expressed as means and standard deviations. For qualitative variables, we chose to use Fisher's exact test or χ^2^ test depending on data. Quantitative variables were compared by *t-*test and ranked data was analyzed by Wilcoxon rank-sum tests or Spearman correlation test. In this study, *P* ≤ 0.05 was considered as statistically significant.

## Results

From December 2018 to March 2019, 156 patients were diagnosed with severe influenza infection in West China Hospital of Sichuan University, of which 72 patients matched our IPA definition. Biopsies is necessary for Proven IPA. All patients in our study lacked biopsy examination due to severe illness, so all IPA cases in our study belonged to probable IPA or possible IPA. All the data of severe influenza patients with and without aspergillosis co-infection are summarized in Table 1.

**Table 1:** Comparisons between severe influenza patients with and without aspergillosis.

	**Without aspergillosis** ***n =* 84**	**With** **aspergillosis** ***n =* 72**	**p Value**
**PATIENTS CHARACTERISTICS, NUMBER (%)**			
Sex (men)	57/84 (67.9%)	50/72 (69.4%)	0.831
Age, mean ± SD	59.1 ± 17.0	58.1 ± 16.0	0.695
Age ≥ 65years	35/84(41.7%)	24/72 (33.3%)	0.285
Underlying disease^*^			0.567
Without underlying disease	25/84 (29.8%)	21/72 (29.2%)	
One type of underlying disease	33/84 (39.3%)	24/72 (33.3%)	
≥ 2 types of underlying disease	26/84 (31.0%)	27/72 (37.5%)	
Immunosuppressant use before admission	1/84 (1.2%)	2/72 (2.8%)	0.596
Steroids use before admission	6/84 (7.1%)	10/72 (13.9%)	0.166
**SEVERITY OF DISEASE, NUMBER (%)**			
Respiratory failure			
Oxygenation index <300 Oxygenation index <100	48/60 (80.0%) 9/60 (15.0%)	62/66 (93.9%)14/66 (21.2%)	0.019 0.367
Mechanical ventilation^Δ^	36/84 (42.9%)	53/72 (73.6%)	0.001
ICU admission	28/84 (33.3%)	46/72 (63.9%)	0.000
Mortality	19/84 (22.6%)	38/72 (52.8%)	0.000
**CLINICAL CHARACTERISTICS**			
Oneset symptoms, number (%)			
Fever	53/84 (63.1%)	53/72 (73.6%)	0.161
Hemoptysis	13/84 (15.5%)	4/72 (5.6%)	0.047
Wheezing	18/84 (21.4%)	36/72 (50.0%)	0.000
LABORATORY EXAMINATIONS, MEAN ± SD			
WBCs, 10^9^/L	9.1 ± 4.8	9.8 ± 5.0	0.540
Neutrophiles, 10^9^/L	7.7 ± 4.6	8.3 ± 4.6	0.371
PCT, ng/mL	1.9 ± 5.9	3.4 ± 6.7	0.182
CRP, mg/L	78.5 ± 75.2	117.6 ± 88.1	0.017
IL-6, μg/L	69.9 ± 100.0	133.5 ± 149.2	0.021
ALB, g/L	34.8 ± 5.7	31.0 ± 5.3	0.000
CEA, ng/mL	11.2 ± 34.8	7.1 ± 5.8	0.553
Lymphocytes, 10^9^/L	0.9 ± 0.8	0.8 ± 0.6	0.249
CD4^+^ T cells, cell/μL	270.8 ± 187.5	275.8 ± 233.0	0.926
RADIOLOGICAL EXAMINATIONS, NUMBER (%)			
Consolidation	18/80 (22.5%)	24/71 (33.8%)	0.122
GGO	54/80 (67.5%)	31/71 (43.7%)	0.003
Nodule	5/80 (6.3%)	13/71 (18.3%)	0.022
Cavitation	3/80 (3.8%)	3/71 (4.2%)	1.000
MYCOLOGICAL EXAMINATIONS, NUMBER (%)			
Influenza type			
Influenza A	83/84 (98.8%)	69/72 (95.8%)	
A, H1N1	67/84 (79.8%)	66/72 (91.7%)	0.037^a^
A, H3N2	2/84 (2.4%)	1/72 (1.4%)	1.000^b^
A, undifferentiated 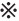	14/84 (16.7%)	2/72 (2.8%)	
Influenza B	1/84 (1.2%)	3/72 (4.2%)	0.336^c^
Sputum cultivation (bacterium)			0.040
Sputum cultivation-negative	43/73 (58.9%)	31/71 (43.7%)	
One type of bacterium in sputum	23/73 (31.5%)	26/71 (36.6%)	
≥2 types of bacterium in sputum	7/73 (9.6%)	14/71 (19.7%)	
Bacterium strain in sputum			
*Baumann/Acinetobacter haemolyticus*	16/73 (21.9%)	27/71 (38.0%)	0.035
*Seudomonas aeruginosa*	2/73 (2.7%)	10/71 (14.1%)	0.014
*Klebsiella pneumoniae*	7/73 (9.6%)	5/71 (7.0%)	0.580
*Escherichia coli*	3/73 (4.1%)	2/71 (2.8%)	1.000
Blood cultivation-positive (bacterium)	4/25 (16.0%)	9/47 (19.1%)	0.764

a *Comparison of H1N1 and other subtypes (H3N2 and B)*.

b *Comparison of H3N2 and other subtypes (H1N1 and B)*.

c *Comparison of influenza A and influenza B*.

* *Underlying disease: chronic obstructive pulmonary disease (COPD), diabetes mellitus (DM), hypertension, end-stage renal disease, liver cirrhosis, solitary cancer, autoimmune disorders and hematological disease*.

Δ *Mechanical ventilation: both non-invasion ventilation and tracheal intubation*.

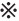
*Influenza A, undifferentiated: the examinations they underwent were unable to identify the subtypes of influenza A*.

*ICU, intensive care unit; WBC, white blood count; PCT, procalcitonin; CRP, C-reactive protein; IL-6, interleukin 6; ALB, serum albumin; CEA, carcino-embryonic antigen; GGO, ground-glass opacity*.

### Clinical Characteristics of Severe Influenza Patients With and Without Aspergillosis

No significant differences were observed in terms of age, male sex, underlying diseases, immunosuppressive agents, and steroids use prior to hospitalization between severe influenza patients with and without aspergillosis (*P* > 0.05).

Fever was the main symptom of severe influenza, but no difference was observed in patients with and without aspergillosis in term of fever (63.1 vs. 73.6%, *P* = 0.161). More wheezing was detected in patients with aspergillosis than those without it (50.0 vs. 21.4%, *P* = 0.000). More patients with hemoptysis were found in patients with influenza alone (15.5 vs. 5.6%, *P* = 0.047).

Both serum CRP level (117.6 ± 88.1 vs. 78.5 ± 75.2, *P* = 0.017) and IL-6 level (133.5 ± 149.2 vs. 69.9 ± 100.0, *P* = 0.021) of severe influenza patients with aspergillosis were greatly higher than those without aspergillosis. However, serum PCT level, WBC, and Neutrophile count were not found to be different between two groups. Moreover, the aspergillosis group had a significant lower ALB level (34.8 ± 5.7 vs. 31.0 ± 5.3, *P* = 0.000) than patients with influenza alone.

GGO pattern was the most common abnormal pulmonary imaging characteristic and patients with influenza alone presented with GGO pattern (67.5 vs. 43.7%, *P* = 0.003) more often than those with aspergillosis. Compared to pulmonary imaging characteristics of patients with influenza alone, more nodule pattern (18.3 vs. 6.3%, *P* = 0.022) were observed in patients with aspergillosis.

Influenza A was the most common influenza subtype identified in both patients with and without aspergillosis. More H1N1 was shown in severe influenza patients with aspergillosis than those without aspergillosis (91.7 vs. 79.8%, *P* = 0.037).

The overall distribution of bacterium in sputum was different between the two groups (*P* = 0.040). Among severe influenza patients with aspergillosis, *Baumann/Acinetobacter haemolyticus* (38.0%) was the bacterium most frequent identified in sputum, followed by *Pseudomonas aeruginosa* (14.1%) and *Klebsiella pneumoniae* (7.0%). As for severe influenza patients without aspergillosis, *Baumann/Acinetobacter haemolyticus* (21.9%), *Klebsiella pneumoniae* (9.6%), and *Esecherichia coli* (4.1%) were the three most common bacteria identified in sputum. The result of bacteria species distribution in sputum showed that more *Baumann/Acinetobacter haemolyticus* (38.0 vs. 21.9%, *P* = 0.035) and *Seudomonas aeruginosa* (14.1 vs. 2.7%, *P* = 0.014) were shown in patients with aspergillosis. The result of blood bacterial cultivation showed that *Baumann/Acinetobacter haemolyticus* was the most common bacteria species identified in both patients with aspergillosis (8.7%) and patients with influenza alone (8.0%) (Supplementary Materials).

### Comparison of Outcome Parameters in Severe Influenza Patients With and Without Aspergillosis

Compared with patients without aspergillosis, more patients with type I respiratory failure (oxygenation index < 300) were observed in severe influenza with aspergillosis (80.0 vs. 93.9%, *P* = 0.019). Severe influenza patient with aspergillosis required mechanical ventilation (both non-invasion ventilation and tracheal intubation) (73.6 vs. 42.9%, *P* = 0.001) and ICU admission (63.9 vs. 33.3%, *P* = 0.000) more often than did patients without it. Moreover, patients with aspergillosis following influenza had a significantly higher mortality than those without it (52.8 vs. 22.6%, *P* = 0.000).

### Prognostic Factors of Severe Influenza Patients With Aspergillosis

Thirty-four patients (47.2%) were alive and 38 patients (52.8%) had died among influenza associated aspergillosis cases. In order to find out factors related with poor prognosis of influenza associated aspergillosis cases, we further divided aspergillosis group into death group and survive group. Result showed more patients with oxygenation index < 100 were observed in death group than survive group (31.6 vs. 7.1%, *P* = 0.016). The rates of mechanical ventilation (92.1 vs. 52.9%, *P* = 0.000) and ICU admission (89.5 vs. 35.3%, *P* = 0.000) in death group were significantly higher than survive group.

No difference in terms of age, gender, underlying diseases, immunosuppressive agents and steroids use was observed between death group and survive group (Table 2). A significantly reduction of CD4^+^ T cells count were observed in death group (189.2 ± 135.3 vs. 367.1 ± 280.0, *P* = 0.022). Further subgroup analysis revealed a significantly higher mortality in aspergillosis group with CD4^+^ T cells count < 200 cell/μL (60.7 vs. 30.6%, *P* = 0.023) (Supplementary Materials). A higher serum WBCs (10.9 ± 5.0 vs. 8.4 ± 3.3, *P* = 0.016), Neutrophiles (9.5 ± 5.0 vs. 7.0 ± 3.8, *P* = 0.023) and PCT (8.6 ± 15.9 vs. 1.2 ± 2.1, *P* = 0.009) level were observed in death group than survive group. Moreover, patients in dead group had a lower ALB level (29.7 ± 5.3 vs. 32.4 ± 4.9, *P* = 0.025) and higher CEA level (10.7 ± 6.8 vs. 4.3 ± 2.7, *P* = 0.008) than those alive.

**Table 2:** Comparison between death group and survive group in severe influenza with aspergillosis.

	**Death group** ***n =* 38**	**Survive group *n =* 34**	***P*-value**
**PATIENTS CHARACTERISTICS, NUMBER (%)**			
Sex(men)	24/38 (63.2%)	26/34 (76.5%)	0.221
Age, mean ± SD	59.3 ± 15.9	56.7 ± 16.3	0.498
Age ≥ 65years	13/38 (34.2%)	11/34 (32.4%)	0.867
Underlying disease^*^			0.273
Without underlying disease	9/38 (23.7%)	12/34 (35.3%)	
One type of underlying disease	13/38 (34.2%)	11/34 (32.4%)	
≥ 2 types of underlying disease	16/38 (42.1%)	11/34 (32.4%)	
Immunosuppressant use before admission	2/38 (5.3%)	0/34 (0.0%)	0.495
Steroids use before admission	8/38 (21.1%)	2/34 (5.9%)	0.090
Cumulative doses of steroids before IPA^#^	32.73 ± 65.92	48.77 ± 103.22	0.113
IPA diagnosis			0.761
Proven IPA	0/38 (0.0%)	0/34 (0.0%)	
Probable IPA	35/38 (92.1%)	33/34 (97.1%)	
Possible IPA	3/38 (7.9%)	1/34 (2.9%)	0.617^d^
Empirical anti-fungal therapy	27/38 (71.1%)	29/34 (85.3%)	0.147
**SEVERITY OF DISEASE, NUMBER (%)**			
Respiratory failure			
Oxygenation index <300 Oxygenation index <100	36/38 (94.7%) 12/38 (31.6%)	26/28 (92.9%)2/28 (7.1%)	1.000 0.016
Mechanical ventilation^Δ^	35/38 (92.1%)	18/34 (52.9%)	0.000
ICU admission	34/38 (89.5%)	12/34 (35.3%)	0.000
**CLINICAL CHARACTERISTICS**			
ONESET SYMPTOMS, NUMBER (%)			
Fever	27/38 (71.1%)	26/34 (76.5%)	0.603
Hemoptysis	0/38 (0.0%)	4/34 (11.8%)	0.045
Wheezing	20/38 (52.6%)	16/34 (47.1%)	0.637
LABORATORY EXAMINATIONS, MEAN ± SD			
WBCs, 10^9^/L,	10.9 ± 5.0	8.4 ± 3.3	0.016
Neutrophiles, 10^9^/L	9.5 ± 5.0	7.0 ± 3.8	0.023
PCT, ng/mL	8.6 ± 15.9	1.2 ± 2.1	0.009
CRP, mg/L	138.1 ± 84.1	95.5 ± 88.5	0.081
IL-6, μg/L	170.5 ± 90.6	174.3 ± 102.0	0.087
ALB, g/L	29.7 ± 5.3	32.4 ± 4.9	0.025
CEA ng/mL	10.7 ± 6.8	4.3 ± 2.7	0.008
Lymphocytes, 10^9^/L	0.8 ± 0.8	0.8 ± 0.4	0.906
CD4^+^ T cells, cell/μL	189.2 ± 135.3	367.1 ± 280.0	0.022
RADIOLOGICAL EXAMINATIONS, NUMBER (%)			
Consolidation	16/37 (43.2%)	8/34 (23.5%)	0.078
GGO	16/37 (43.2%)	15/34 (44.1%)	0.941
Nodule	5/37 (13.5%)	8/34 (23.5%)	0.276
Cavitation	0/37 (0.0%)	3/34 (8.8%)	0.065
MYCOLOGICAL EXAMINATIONS, NUMBER (%)			
Influenza type			0.456
Influenza A	37/38 (97.4%)	32/34 (94.1%)	
A, H1N1	35/38 (92.1%)	31/34 (91.2%)	1.000^a^
A, H3N2	0/38 (0.0%)	1/34 (2.9%)	0.472^b^
A, undifferentiated 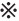	2/38 (5.3%)	0/34 (0.0%)	
Influenza B	1/38 (2.6%)	2/34 (5.9%)	0.599^c^
Sputum cultivation(bacterium)			0.491
Sputum cultivation-negative	14 /37 (37.8%)	17/34 (50.0%)	
One type of bacterium in sputum	16/37 (43.2%)	10/34 (29.4%)	
≥ 2 types of bacterium in sputum	7 /37 (18.9%)	7/34 (20.6%)	
Bacterium strain in sputum			
*Baumann/Acinetobacter haemolyticus*	17/37 (45.9%)	10/34 (29.4%)	0.152
*Seudomonas aeruginosa*	3/37 (8.1%)	7/34 (20.6%)	0.131
*Klebsiella pneumoniae*	3/37 (8.1%)	2/34 (5.9%)	1.000
*Escherichia coli*	1/37 (2.7%)	1/34 (2.9%)	1.000
Blood cultivation-positive (bacterium)	7/26 (26.9%)	2/21 (9.5%)	0.160
Fungus inspection			
GM (serum)-positive	22/34 (64.7%)	19/33 (57.6%)	0.729
GM (serum) index			0.564
0.5–1.0	10/34 (29.4%)	9/33 (27.3%)	
1.0–1.5	2/34 (5.9%)	1/33 (3.0%)	
>1.5	10/34 (29.4%)	9/33 (27.3%)	
GM (BAL)-positive	12/12 (100.00%)	11/12 (91.7%)	1.000
Fungal cultivation-positive	19/33 (57.6%)	18/34 (52.9%)	0.703

a *Comparison of H1N1 and other subtypes (H3N2 and B)*.

b *Comparison of H3N2 and other subtypes (H1N1 and B)*.

c *Comparison of influenza A and influenza B*.

d *Comparison of probable IPA and possible IPA*.

* *Underlying disease: chronic obstructive pulmonary disease (COPD), diabetes mellitus (DM), hypertension, end-stage renal disease, liver cirrhosis, solitary cancer, autoimmune disorders and hematological disease*.

# *Cumulative doses of steroids before IPA: the steroid doses were converted to prednisone doses*.

Δ *Mechanical ventilation: both non-invasion ventilation and tracheal intubation*.

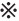
*Influenza A, undifferentiated: the examinations they underwent were unable to identify the subtypes of influenza A*.

*IPA, invasive pulmonary aspergillosis; ICU, intensive care unit; WBC, white blood count; PCT, procalcitonin; CRP, C-reactive protein; IL-6, interleukin 6; ALB, serum albumin; CEA, carcino-embryonic antigen; GGO, ground-glass opacity; GM, galactomannan; BAL, bronchoalveolar lavage fluid*.

Pulmonary radiological examinations showed no significant difference between death group and survive group (Table 2). *Bowman/Acinetobacter haemolyticus* was the most frequent bacterium isolated in both sputum (17/37, 45.9%) and blood (3/26, 11.5%) in death group. No differences in the positive detection rate of GM test (serum and BAL) and fungal cultivation were observed between the death and survive group. Our data did not show obvious correlation between serum GM index and mortality in severe influenza patients with aspergillosis (Table 2).

## Discussion

Analysis of clinical data of severe influenza patients showed that there were no significant correlation between patient characteristics (including age, gender, underlying diseases, immunosuppressive agents, and steroids use) and the incidence and mortality of influenza associated aspergillosis. H1N1 was the major influenza subtype of severe influenza patients with aspergillosis, and patients with more serious inflammatory response and respiratory failure were observed in influenza associated aspergillosis. Aspergillosis co-infection following severe influenza could lead to a significant increase in mortality, which was associated with more severe bacterial infection, respiratory failure, as well as poorer immunity and nutritional status.

Contrary to previous researches (1, 3–5), we did not find the poor baseline characteristic of patients and decreased CD4^+^ T cells count followed by influenza had any impact on the occurrence of aspergillosis co-infection. But severe influenza patients with aspergillosis mostly belonged to the H1N1 category and accompanied with higher inflammatory level represented by CRP and IL-6. These data suggested an association between aspergillus invasion and influenza virus infection. Although the pathogenesis of aspergillosis in the setting of severe influenza have not to be defined yet, it is generally recognized that influenza may promote IPA *via* breakdown of bronchial mucosa and disruption of mucociliary clearance (22), which was related with increased risk of aspergillosis. In comparison with seasonal influenza virus, H1N1 virus causes more damage to the airway epithelial cells (21). But much more importantly, the pulmonary severe inflammatory response induced by influenza invasion played a more crucial role in pathogenesis of aspergillosis co-infection and also was the major cause of ARDS in severe influenza patients. A biphasic immunological response to severe infection (an early hyper-inflammatory phase followed by an anti-inflammatory response, leading to a hypo-inflammatory state, which is called compensatory anti-inflammatory response syndrome) was reported to make immunocompetent hosts vulnerable to bacteria and fungi co-infection (23). IL-6 and CRP were the synthesis of pro-inflammatory cytokines released from the epithelial cells invaded by influenza and resulted in further alveolar damage according to enhance phagocytosis of immune cells and activate a series of innate immunoresponses (24, 25). The significant up-regulated IL-6 and CRP level were observed in severe influenza (especially H1N1category) and were linked to severe tissue and organ damage and to the likelihood of developing more severe clinical manifestations, respiratory failure and complications, as well as poorer outcome (26). Our data also showed rapidly increased secretion of IL-6 and CRP in severe influenza patients with aspergillosis than those with influenza alone. These suggested that the occurrence of aspergillosis co-infection was related with influenza virus attacks and resulting pulmonary excessive inflammatory response in immunocompetent hosts.

Bacterial superinfection was generally considered to be related to a higher incidence and mortality of influenza associated aspergillosis (27). Our result also revealed increased bacterial infection indicators (e.g., WBC, Neutrophiles counts, and PCT) in both severe influenza patients with and without aspergillosis. Further analysis indicated no impact of bacterial infection on the incidence of influenza associated aspergillosis. But a higher level of bacterial infection (represented by WBC, Neutrophiles counts, and PCT level on admission) accompanied by a lower oxygenation index was observed to be associated with higher mortality of these severe influenza patients with aspergillosis. As we known, viral infection can result in a decreased CD4^+^ T cells, which actually plays an essential role in the defense against fungal infections (5). All the patients in our study had no history of immunodeficiency before admission, so decreased CD4^+^ T cells count was regarded as a subsequent change of viral infection. Although decreased CD4^+^ T cells in severe influenza patients was thought to be associated with aspergillus infection in a study with small sample (28), our data do not support the association between decreased CD4^+^ T cells and the occurrence of influenza associated aspergillosis. But reduction of CD4^+^ T cells was proved to be associated with higher mortality of severe influenza patients with aspergillosis, especially in patients with CD4^+^ T cells count < 200 cell/μL. In our study, IPA co-infection increased overall mortality of severe influenza patients from 22.64 to 52.78%. Based on previous studies and our data, we considered that the high mortality of severe influenza associated aspergillosis was mainly related with severe respiratory failure secondary to mixed infection (including viral, bacteria, and aspergillosis) and failure to recovery of immunologic dissonance induced by viral invasion.

In our study, 77.27% diagnosis of IPA co-infection was confirmed within 3 days after influenza diagnosis. Therefore, it is essential to emphasize positive searching for evidence of fungal co-infections in early stage. Severe influenza with IPA in immunocompetent hosts did not have classical radiographic findings (e.g., cavitary lesions, halo, or crescent) (1, 9, 20), the most important clinical diagnosis method of IPA still depends on fungal screening, including GM tests and fungal cultivation. Compared with serum GM test sensitivity of 33% among IPA patients in ICU without classical host risk factors (29), our data suggested that serum GM tests of influenza associated aspergillus cases revealed superior performance with positive rate of 61.2%. So serum GM test can be recommended as an active monitoring method for severe influenza patients at high risk of fungal co-infection, especially for patients unable to obtain BAL GM test due to serious illness or primary hospitals without condition to conduct bronchoalveolar lavage examination. Serum GM test would be particularly important to provide proof for early diagnosis.

## Conclusion

Aspergillosis co-infection in severe influenza patients can lead to a significant increased mortality, and the high mortality was associated severe respiratory failure secondary to mixed infection (including viral, bacteria, and aspergillosis). Active and effective controlling infection, improving immunity, and nutrition status were the key to lowering the morality of influenza associated aspergillosis. Aspergillus co-infection in severe influenza patients was related with influenza subtype and excessive pulmonary inflammatory response, but had no relation with age, gender, underlying diseases, CD4^+^ T cells count, immunosuppressive agents, and steroids use. Therefore, for all severe influenza patients with suspicious fungal signs and symptoms, clinicians should take initiative to search for microbiological evidence to avoid delayed and missed diagnosis of aspergillosis co-infection.

## Data Availability Statement

The original contributions presented in the study are included in the article/Supplementary Materials, further inquiries can be directed to the corresponding author/s.

## Ethics Statement

Ethical review and approval was not required for the study on human participants in accordance with the local legislation and institutional requirements. Written informed consent for participation was not required for this study in accordance with the national legislation and the institutional requirements. Written informed consent was not obtained from the individual(s) for the publication of any potentially identifiable images or data included in this article.

## Author Contributions

YSD made substantial contributions to the study design, data acquisition, analysis, or interpretation and drafting the final manuscript. XMO designed the study. YSC, XYO, and BML contributed to the data collection and completed the statistical analysis. All authors read and approved the final manuscript.

## Conflict of Interest

The authors declare that the research was conducted in the absence of any commercial or financial relationships that could be construed as a potential conflict of interest.
