# Supplementary material for: Severe Influenza With Invasive Pulmonary Aspergillosis in Immunocompetent Hosts: A Retrospective Cohort Study
Source: Front Med (Lausanne). 2021 Jan 18;7:602732. doi: 10.3389/fmed.2020.602732 (PMC7848171; doi:10.3389/fmed.2020.602732)
Supplement: Supplementary file 1 [file Data_Sheet_1.DOCX]

**Bacterium strain in sputum and blood between severe influenza patients with and without aspergillosis**

|  | Without  aspergillosis n=84 | With  aspergillosis n=72 |
| --- | --- | --- |
|  |  |  |
|  |  |  |
| Bacterium strain in sputum | | |
| Baumann/Acinetobacter haemolyticus | 16/73 (21.9%) | 27/71 (38.0%) |
| Seudomonas aeruginosa | 2/73 (2.7%) | 10/71 (14.1%) |
| Klebsiella pneumoniae | 7/73 (9.6%) | 5/71 (7.0%) |
| Escherichia coli | 3/73 (4.1%) | 2/71 (2.8%) |
| Staphylococcus aureus | 2/73 (2.7%) | 2/71 (2.8%) |
| Acinetobacter haemolyticus | 0/73 (0.0%) | 2/71 (2.8%) |
| G-bacteria | 1/73 (1.4%) | 1/71 (1.4%) |
| Mannitol ralstonia | 1/73 (1.4%) | 1/71 (1.4%) |
| Enterobacter cloacae | 1/73 (1.4%) | 1/71 (1.4%) |
| Burkholderia | 1/73 (1.4%) | 1/71 (1.4%) |
| Haemophilus influenzae | 0/73 (0.0%) | 1/71 (1.4%) |
| Staphylococcus epidermidis | 0/73 (0.0%) | 1/71 (1.4%) |
| Stenotrophomonas maltophilia | 0/73 (0.0%) | 1/71 (1.4%) |
| Corynebacterium striata | 0/73 (0.0%) | 1/71 (1.4%) |
| Pneumocystis pneumoniae | 0/73 (0.0%) | 1/71 (1.4%) |
| Enterococcus excreta | 0/73 (0.0%) | 1/71 (1.4%) |
| Hospital acinetobacter | 1/73 (1.4%)  1 (1.4%) | 0/71 (0.0%) |
| Bacterium strain in blood | | |
| Baumann/Acinetobacter haemolyticus | 2/25 (8.0%) | 4/46 (8.7%) |
| Seudomonas aeruginosa | 0/25 (0.0%) | 1/46 (2.2%) |
| Klebsiella pneumoniae | 1/25 (4.0%) | 1/46 (2.2%) |
| Escherichia coli | 0/25 (0.0%) | 1/46 (2.2%) |
| Staphylococcus aureus | 0/25 (0.0%) | 1/46 (2.2%) |
| Staphylococcus | 1/25 (4.0%) | 0/46 (0.0%) |
| Pneumococci | 1/25 (4.0%) | 0/46 (0.0%) |
| Enterococcus excreta | 0/25 (0.0%) | 1/46 (2.2%) |
| Staphylococcus epidermidis | 0/25 (0.0%) | 1/46 (2.2%) |

## Bacterium strain in sptumn and blood between death group and survive group in severe influenza with aspergillosis

|  | Death group n=38 | Survive group n=34 |
| --- | --- | --- |
|  |  |  |
|  |  |  |
| Bacterium strain in sptumn | | |
| Baumann/Acinetobacter haemolyticus | 17/37(46.0%) | 10/34(29.4%) |
| Seudomonas aeruginosa | 3/37 (8.1%) | 7/34 (20.6%) |
| Klebsiella pneumoniae | 3/37 (8.1%) | 2/34 (5.9%) |
| Escherichia coli | 1/37 (2.7%) | 1/34 (2.9%) |
| Staphylococcus aureus | 1/37 (2.7%) | 1/34 (2.9%) |
| Acinetobacter haemolyticus | 2/37 (5.4%) | 2/34 (5.9%) |
| G-bacteria | 1/37 (2.7%) | 0/34 (0.0%) |
| Corynebacterium striata | 1/37 (2.7%) | 0/34 (0.0%) |
| Pneumocystis pneumoniae | 1/37 (2.7%) | 0/34 (0.0%) |
| Stenotrophomonas maltophilia | 0/37 (0.0%) | 1/34 (2.9%) |
| Bacterium strain in blood | | |
| Baumann/Acinetobacter haemolyticus | 3/26 (11.5%) | 1/21 (4.8%) |
| Seudomonas aeruginosa | 0/26 (0.0%) | 1/21 (4.8%) |
| Klebsiella pneumoniae | 1/26 (3.9%) | 0/21 (0.0%) |
| Escherichia coli | 1/26 (3.9%) | 0/21 (0.0%) |
| Staphylococcus aureus | 0/26 (0.0%) | 1/21 (4.8%) |
| Enterobacter cloacae | 1/26 (3.9%) | 0/21 (0.0%) |
| Enterococcus excreta | 1/26 (3.9%) | 0/21 (0.0%) |

**Subgroup anlysis of CD4+T cells on hospital admission**

|  | CD4+T cells on hospital admission | | P value |
| --- | --- | --- | --- |
|  | ≥200 cells/μL | <200 cells/μL |  |
| IPA morbidity,number(%) | 20/36(55.6%) | 17/28(60.7%) | 0.804 |
| Mortality,number(%) | 11/36(30.6%) | 17/28(60.7%) | 0.023 |
